# Selection of Anesthesia for Oral Surgical Operations, and the Roentgen Ray as an Aid in Diagnosis*Extract from paper published in March *Cosmos* with illustrations, page 209.

**Published:** 1918-05

**Authors:** Kurt H. Thoma


### SELECTION OF ANESTHESIA FOR ORAL SURGICAL OPERATIONS, AND THE ROENTGEN RAY AS AN AID IN DIAGNOSIS*

BY KURT H. THOMA, D.M.D.

Before undertaking an operation in the mouth it is important to see the patient for the purpose of making an accurate diagnosis and selecting the most suitable form of anesthesia. The two principal types from which to choose are local and general.

For operations to be performed in the office, where it is difficult to prepare the patient properly, local anesthesia is preferable. It has the advantage of freeing the region of the operation from the presence of the

*Extract from paper published in March *Cosmos* with illustrations, page 209.

anesthetist and his appliances; also it eliminates the dangers attending general anesthesia, that is, swallowing and inspiration of blood and pus. Then, too, the absence of vomiting after the operation is a point in its favor, as there is no disintegration of the wound by the vomitus. Fruthermore, with local anesthesia the cooperation of the patient is possible, and the hemostatic action of the injection makes the field almost bloodless, both of which factors facilitate the work of the operator.

Notwithstanding these great advantages we must nevertheless select our cases for local anesthesia. Children are usually poor subjects, although very intelligent older children prove to be exceptions in some cases. Among adults we have those who will not consent to anything but a general anesthetic, but for many patients a general anesthetic is contraindicated. In such cases danger can be avoided by the use of local anesthesia.

The excitable, nervous, or timid patient who dreads knowing what is going on and is afraid of the instrumentation can sometimes be taken care of by preanesthetic medication, such as bromural, veronal, or morphia, but as a rule does better under a general anesthetic.

The majority of dental and oral surgical operations can be performed with the intraoral method of local anesthesia, while the extraoral method is indicated for all extensive cases where a large area is to be anesthetized, and where the use of the intraoral method would necessitate injection into pathological tissue.

The most commonly used intraoral methods are the zygomatic, infraorbital, palatal, and pterygomandibular injections. The principal extraoral injections are the infraorbital, maxillary, and mandibular.

If general anesthesia is used in oral surgery we have the choice between nitrous oxid and oxygen, ether, and a combination of the two. Nitrous oxid and oxygen has been used so long for short operations that it needs no further recommendation. However, it does not always insure an uninterrupted operation, even if administered by an experienced anesthetist, especially if the operation is of long duration. I therefore prefer ether given by one of the newer methods, as a perfect continuous anesthesia can be obtained with it. After anesthesia is induced by nitrous oxid and oxygen the patient ordinarily receives ether applied with a cone, and when sufficiently relaxed, ether vapors are administered by a pump into the pharynx, either from a tube extending back into the mouth or by the insertion of a nasopharyngeal tube, which extends into the nasopharynx.

Another way is the intratracheal method, which is very satisfactory, but more difficult in its application. The tubes are passed through each nostril into the nose and pharynx and from there into the trachea, the ether being given through a funnel filled with gauze, and connected to the intratracheal tubes by a larger piece of rubber tubing. The patient inhales air saturated with ether vapors, and care must be taken that no liquid ether is allowed to enter the lungs.

Large oral surgical operations should only be undertaken in a hospital, and never in an office, as it is not fair to give a patient general anesthesia without proper preparation and after-care. As many of our operations require special apparatus, it is the general tendency to undertake too much in the office. In order to overcome this I have equipped for my purpose an operating room for oral surgical operations at one of our private hospitals, and find that patients are very appreciative of the care they receive before and after the operation, especially if they come from out of town.

In operations performed under a general anesthetic it is advisable to use an aspirator to take up the saliva and blood, and the conditions can also be improved by employing a combination of local and general anesthesia.

This not only does away with the surgical shock, but by decreasing the amount of bleeding clears the field of operation.

Quite as important as selecting the anesthetic is the making of a correct diagnosis. The roentgen ray is the most important means of making an examination, and in a great many cases it gives exact and accurate information. It should not, however, be entirely relied upon, and a study of the history and symptoms of the case should not be overlooked.

For lesions of the jaws and unerupted third molar teeth extraoral plates are preferable. The small area covered by a film is quite often insufficient and misleading in its information, especially if a tooth is misplaced or if the lesion is extensive. The object to be roentgenographed should not only have its entire outline shown, but an idea should be given of its proximity to adjacent structures, and enough of the surrounding normal tissue should appear in the plate to make possible a contrast between it and the parts affected by disease.

### DIFFICULT EXTRACTIONS, IMPACTIONS, AND EXOSTOSES

The extraction of impacted teeth is one of the most common oral surgical operations, and a good roentgen picture is absolutely necessary to plan the procedure by which it can be removed with the least injury to the remaining tissue. I prefer extraoral plates for impacted third molars, but occasionally a film will suffice. However, a negative finding on the film should always be verified by a plate, unless there is a history that the tooth has been removed. Quite frequently third molars are situated so far away from their normal positions that they would not come into the intraoral picture, and this might lead to the conclusion that the tooth was congenitally absent.

Teeth which have been devitalized for a long time often show an enlarged condition at their apices, which is known as exostosis. This enlargement is due to hypercementosis caused by the continued chronic inflammation, which stimulates the cementoblasts to form new layers of cement at the part where the lesion occurs. Such teeth, especially if located in the lower jaw, are difficult to extract, and, as a rule can only be removed after a considerable portion of the alveolar process has been dissected away. There are many reasons why it is advantageous to diagnose such conditions by means of roentgenograms. Preparation can be made, *e. g.*, for an operation that requires more instrumentation than a simple extraction, and there is no danger of the patient getting the idea, as sometimes happens, that the operator fractured the tooth on account of unskilfulness, and then had to resort to a long, tedious procedure, which may make him resentful and unwilling to pay an adequate fee. The operator is also able to choose an anesthetic which is sufficiently lasting.

### BLIND ABSCESSES

The development of the roentgen ray for dentistry made us realize the conditions which may be caused by devitalized teeth in the alveolar process and the jaws. The bone surrounding the roots of such teeth is almost always more or less involved and displaced by granulation tissue of infectious nature. This loss of bone shows as a dark area in the roentgenogram, and it is important to realize that such conditions start and may grow rapidly worse without giving any discomfort to the patient or obvious signs of disease. If teeth are extracted without considering these infections of the bone, the condition may continue to exist in the mouth for many years and be a source from which absorption of pus may occur.

It is therefore imperative to see whether the tooth to be extracted is complicated by the condition commonly called a granuloma or blind abscess, and to find out its size and its relation to important adjacent structures, such as the mandibular canal or the antrum of Highmore.

In a case where the blind abscess is quite extensive the necessity of properly opening into such an area and curetting it is quite evident.

### MAXILLARY SINUSITUS CAUSED BY CONDITIONS OF THE TEETH

Maxillary sinusitis, in its acute suppurative, or chronic form, is frequently caused by infections from the teeth. According to Brophy, about 75 per cent. of all such cases are due to dental infection. Chronic maxillary sinusitis often occurs without any alarming symptoms in a manner similar to that in which blind abscesses or granulomata are formed, and it is common for such conditions to be discovered during a merely routine examination. This brings out the importance of determining the condition of the maxillary sinuses when large abscesses are found on the upper bicuspid and molar teeth. On the other hand, when a diseased antrum is found, one should always take roentgenograms of the teeth to see if the trouble is associated with disease conditions of these organs.

A patient had pain in the zygomatic and infraorbital regions with discharge from the right nostril. A frontal roentgen plate showed radiopacity of the right antrum. The cause was ascertained by a film, which showed radiolucent areas on two roots of the upper first molar, indicating abscesses.

Another patient was in poor health, and was referred by her dentist for extraction of the upper left molar. After extraction of the tooth a probe could be passed into the antrum. The previously taken films of the teeth showed a large radiolucent area on the roots of the upper first molar. The second molor was devitalized also. A frontal plate taken immediately after the extraction showed radiopacity of the left maxillary sinus and a cyst of the right maxillary sinus.

This patient had been in a run-down condition for some time and had been under a physician's care, but did not improve. The roentgenographic examination of the teeth showed indications of many pus pockets and abscesses in the right upper jaw. The condition of her antrum was questioned, and a frontal plate showed it to be involved on that side.

Mrs. F. K. complained of severe pain in the head. Films of the teeth showed abscesses around the upper first molar, which was the abutment of a bridge. Involvement of the maxillary sinuses seemed possible, and a frontal plate proved this to be the case.

### PERIODONTAL OR RADICULAR CYSTS

This type of cyst is of inflammatory nature, and has its origin in an epitheliated granuloma or blind abscess. Exudates which accumulate on the inside cause an expansion of the cyst, which grows at the expense of the bone. They may grow enormously and are, as a rule, associated with a devitalized tooth. At times, however, we find cases where the cyst continues to grow in the jaw independently after the extraction of the tooth. Such a patient remembered that he had had an abscessed tooth on that side extracted about sixteen years before, which must have been the first molar, as this was the only tooth absent. The roentgenogram shows that the tip of one of the roots was left in the jaw and apparently also the abscess sac, which grew into an extremely large cyst. When operating I found the contents of the cyst to be under pressure, and a great deal of pus escaped after the first opening was made to the bone. This case is interesting also from the standpoint of the patient's health having been very poor on account of the condition. He had had some palpitation and dyspnea, and was evidently very anemic. He had been undergoing treatment for about a year when he broke down from nervous collapse. Before the operation the blood count was as follows: Hemoglobin, 85 per cent.; leucocytes, 7000; red count, 5,120,000. One week after the operation his physician reported the count to be—Hemoglobin, 92 per cent.; red count, 5,500,000. Six months later his blood count was again taken, with the following result: Hemoglobin, 93 per cent.; leucocytes, 6000; red count, 5,800,000.

### DENTIGEROUS OR FOLLICULAR CYSTS

Such cysts are not of inflammatory nature, but are of embryonic origin, developing from the tooth follicle of an unerupted impacted or misplaced tooth or tooth germ, and they may contain one or many elementary teeth or well-formed tooth masses. They also grow to tremendous size, and the roentgen picture is one of the best means of making a differential diagnosis. Such a case was referred to me by Dr. H. B. Loder. The patient, a boy twelve years of age, had a swelling on the right side of his face. This was confined to the ascending ramus of the jaw, and had been noticed for nine months, there being also a slight amount of pain. The roentgen picture shows a well-formed cyst containing a number of defined teeth and small foreign bodies, which, being of about the same radiopacity as the teeth, were taken to be indefinite tooth particles.

